# Investigating the Moderating Effect of HIV Status Disclosure on the Link Between Discrimination Experience and Psychological Distress Among People Living with HIV in Japan Infected Through Sexual Contact

**DOI:** 10.1007/s12529-024-10304-3

**Published:** 2024-06-28

**Authors:** Takeshi Miwa, Chihiro Wakabayashi, Kanna Hayashi, Junko Tanuma, Kazuko Ikeda, Yoshiyuki Yokomaku, Yuzuru Ikushima

**Affiliations:** 1 Department of Health Sciences, Saitama Prefectural University, 820 Sannomiya, Koshigaya-shi, Saitama, 343-8540 Japan; 2 Positive Living and Community Empowerment Tokyo (PLACE TOKYO), 4-11-5-403 Takadanobaba, Shinjuku-ku, Tokyo, 169-0075 Japan; 3 Faculty of Health Sciences, Simon Fraser University, Blusson Hall, Room 11300, 8888 University Drive, Burnaby, BC V5A 1S6 Canada; 4 Center Hospital of the National Center for Global Health and Medicine, 1-21-1 Toyama, Shinjuku-ku, Tokyo, 162-8655 Japan; 5 Department of Infectious Diseases & Immunology, Clinical Research Center, National Hospital Organization Nagoya Medical Center, 4-1-1 Sannomaru, Naka-ku, Nagoya, Aichi 460-0001 Japan

**Keywords:** Discrimination experience, Mental health, HIV status disclosure, People living with HIV, Japan

## Abstract

**Background:**

There is a scarcity of research on the potential impact of disclosing HIV status to friends and family in moderating the adverse effects of discrimination on the mental health of people living with HIV (PLWH). This study assessed the experiences of discrimination and HIV status disclosure among PLWH in Japan, and evaluated their potential associations with psychological distress.

**Method:**

Data were derived from a nationwide cross-sectional survey of PLWH conducted in Japan between 2019 and 2020. The interaction effects of HIV-related discrimination and HIV status disclosure on the psychological distress were examined using logistic and linear regression analyses.

**Results:**

The median age of the 804 respondents was 46 years old. Most respondents were male and 85.4% (687/804) identified as homosexuals or bisexuals. A total of 12.7% (102/804) of the respondents reported that they had recently experienced discrimination because of their HIV status. Experience of HIV-related discrimination was independently associated with high psychological distress (adjusted OR 2.02; 95% CI, 1.15–3.57), and HIV status disclosure to friends partially weakened the association between discrimination and the level of psychological distress (regression coefficient −3.115; *p* = 0.004).

**Conclusion:**

While measures that aim to end discrimination remain vital, increasing the opportunities of PLWH to communicate with friends they feel comfortable disclosing their HIV status may also be helpful in protecting their mental health.

## Introduction

Owing to the introduction of combination antiretroviral therapy (ART), the prognosis of people living with HIV (PLWH) has substantially improved, with their life expectancy approaching that of those without HIV infection [1]. However, despite these medical advancements, previous studies have consistently highlighted the prevalence of poor mental health among PLWH [2, 3]. In Japan, research indicates that PLWH exhibit lower mental health-related quality of life compared to the general population [4]. Poor mental health is a significant barrier to ensuring sufficient and sustained care [2], leading to suboptimal adherence and disengagement from care [5].

Among several factors that impact the mental well-being of PLWH, discrimination experience emerges as a paramount concern warranting further investigation. The fear of being discriminated against for being HIV positive has a detrimental impact on mental health by causing depressive symptoms [6]. Similarly, research in Japan identified a notable association between heightened stigmatisation and poor mental health outcomes among PLWH [7]. Despite recent research suggesting that PLWH undergoing treatment with stable viral suppression pose minimal risk of transmitting the virus to sexual partners [8], discrimination against PLWH remains prevalent. Alarmingly, a significant portion of the heterosexual, HIV-uninfected population in Japan expressed strong reservations about HIV-infected individuals marrying their relatives [9]. Such pervasive discriminatory attitudes contribute to heightened levels of anticipated stigma (i.e. expectation of negative treatment due to their HIV status) among PLWH in Japan, surpassing those reported in non-Asian regions [10].

While developing policies and public awareness campaigns that aim to end discrimination remains vital, there is growing recognition that social support can help mitigate the negative impact of discrimination on mental health. A study conducted in Japan found that PLWA lacking sufficient emotional support face heightened risk of depression, underscoring the pivotal role of enhanced social support in safeguarding their mental well-being [11]. Recent research has further elucidated that social support can partially mitigate the deleterious impact of anxiety-mediated HIV stigma on mental health [12]. Furthermore, evidence suggests that engagement in social support groups and bolstered social support may serve to attenuate the association between stigma and suicidal thoughts among PLWH [13]. In addition, PLWH with higher levels of social support were less likely to report experiencing HIV-related stigma [14].

For PLWH, the presence of supportive friends and family members who are aware of their HIV status is an essential component of social support. Research indicates that disclosing one’s HIV status is associated with heightened levels of perceived social support among PLWH [15]. Moreover, HIV status disclosure has been linked to improvements in emotional well-being and treatment adherence among this population [16]. Despite concerns about rejection and stigma from others, research suggests that the positive consequences of disclosing one’s HIV status, such as increased support and care, reduced stress, improved mood, and increased optimism, often outweigh the negative consequences [17]. Furthermore, assisting PLWH in their decision-making process regarding HIV status disclosure has been shown to yield favourable mental health outcomes [18]. However, the level of comfort in sharing one’s HIV status remains notably low among PLWH in Japan, at only 18.7% [10].

Despite much attention being drawn to the potential of HIV status disclosure, the findings pertaining to its role in protecting the mental health of PLWH in Japan from the experience of discrimination remain unexplored. Understanding the implications of HIV status disclosure for PLWH in Japan is crucial for informing support strategies from various stakeholders. Therefore, this study aimed to investigate whether disclosing one’s HIV status could mitigate the negative effects of discrimination on mental health among PLWH, particularly those infected through sexual contact, which represents approximately 90% of all new HIV cases in Japan [19]. Building upon previous findings [6, 7, 12, 13], this study hypothesised that HIV-related discrimination exacerbates the mental health of PLWH, and that HIV status disclosure to people of particular relationships may ameliorate this association.

## Methods

### Study Design

Data were derived from a nationwide cross-sectional survey of PLWH attending eight leading HIV/AIDS care hospitals in Japan (“The Nationwide Health and Lifestyle Survey of People Living with HIV in Japan”), conducted between September 2019 and March 2020. Patients who could not read Japanese were ineligible to participate, because the questionnaire was only in Japanese. In addition, individuals with poor physical health, such as those with cognitive impairment or those hospitalised due to AIDS, were excluded to ensure the reliability of the data and for ethical reasons. The questionnaire had approximately 100 questions, some of which were sensitive and personal, making it inappropriate to ask patients with poor physical health to participate, as this could cause them undue burden or distress.

All the eligible patients were consecutively invited to and informed of the details of the study and provided a written consent before their participation, in the order of their outpatient visits. Those who provided consent were then given a gift voucher worth JPY 500 (approximately 5 USD) and requested to complete an anonymous paper-based questionnaire independently and return it to the investigator via post using the self-addressed envelope provided along with the questionnaire. The recruitment continued until each hospital had distributed their designated number of questionnaires, determined by the size of the hospital. The study protocol was authorised by the ethical review committees of Saitama Prefectural University (No. 30101) and the participating hospitals.

### Participants

Among the 1930 consenting individuals, 1185 (61.4%) returned the questionnaire. The analytic sample was restricted to respondents whose self-reported mode of HIV transmission included sexual contact (homosexual and/or heterosexual). A total of 10.9% (129/1185) of the respondents who reported being infected solely through other means were excluded from the analysis, including needle sharing (9/129), blood coagulation factor products (72/129), blood transfusion (3/129), unknown (43/129), mother-to-child (0/129), and others (2/129). Individuals infected through blood coagulation factor products, representing over half of the excluded respondents, were a key demographic in Japan during the early 1980s when haemophilia patients were infected with HIV through contaminated blood products, leading to scandals and lawsuits [20]. This group has unique needs and characteristics compared to those infected through other means [21], necessitating their exclusion to minimise variability in the analysis. Of the remaining 1056 respondents, 804 with complete answers for relevant questions were included in the analysis.

### Measures

Data on experience of discrimination was derived from a question “Have you recently experienced being placed in a disadvantageous position or felt that you were being discriminated against due to your HIV-positive status?” Although the English translation may not be immediately clear, the original Japanese phrase is commonly used by healthcare professionals in Japan when discussing discrimination experiences with patients. The participating hospitals widely recognised that this phrase would be comprehensible to patients, regardless of their educational background. The response options were presented on a 4-point Likert scale (“Often”, “Sometimes”, “Rarely”, and “Never”). Individuals who responded with “Often” or “Sometimes” were categorised as having experienced discrimination.

Information on HIV status disclosure was captured separately for each relationship using a question: “Have you disclosed your HIV-positive status to people listed below?” The list that followed the question included parent(s), sibling(s), partner(s), HIV-positive friend(s), other friend(s), colleague(s), and boss(es). For each relationship on the list, respondents were required to answer either “Yes”, “No”, or “I don’t have anyone of that relationship”. The respondents’ mental health status was evaluated using the Japanese version of the Kessler 6-Item Psychological Distress Scale (K6 scale) [22]. Respondents were asked to self-report how frequently they had experienced the following symptoms in the past month: (i) felt nervous, (ii) felt hopeless, (iii) felt restless or fidgety, (iv) felt depressed, (v) felt that everything was an effort, and (vi) felt worthless. A value between 0 and 4 was assigned to each response ranging from “None of the time” to “All of the time”, resulting in a total score range from 0 to 24 [23]. In this study, the K6 scale demonstrated high internal consistency, with a Cronbach’s alpha of 0.90, indicating reliable measurement of psychological distress.

Questions regarding socio-demographic characteristics included the following: age, sex assigned at birth, sexual orientation, nationality, highest educational attainment, regular psychiatric visits, current employment status, and willingness to work. Respondents who selected “psychiatric symptoms” and/or “addiction” for the inquiry “Please select any health issues for which you routinely seek medical attention, excluding HIV” were categorised as having regular psychiatric visits. Respondents were categorised as “unemployed” if they selected “no” to the question “During the last week of the previous month, were you engaged in compensated employment?” and as “employed” if they chose “yes, mainly working”, “yes, working while doing housework”, “yes, working while being a student”, or “yes, but was on leave”. Respondents who selected “I do not want to work if possible/I do not intend to work” in response to the question “What is your opinion about working?” were classified as “not willing to work”, while those who chose “I want to work without any particular restrictions” or “I want to work with accommodations to suit my health condition” were classified as “willing to work”.

Questions related to clinical characteristics of HIV included self-reported year of HIV diagnosis, most recent CD4 count, most recent viral load, experience of developing AIDS, instances of forgetting to take antiretroviral medications in the past month, whether the individual had ever made excuses to hide their HIV status, whether they had ever pretended to be HIV-negative, and awareness of the concept that “Undetectable=Untransmittable (U=U)”. For data on instances of forgetting to take antiretroviral medications, respondents who answered “Never” to the question “In the past month, approximately how often have you forgotten to take your antiretroviral medication (i.e., missed a dose for more than 24 hours)?”, were categorised as “no”. On the other hand, those who answered “Almost every day”, “Two to few times a week”, “Once a week”, “Once in two weeks”, or “Once a month” were categorised as “yes”. Respondents who answered “I know” to the question “Are you aware that individuals who have maintained an undetectable viral load of HIV for a period of six months or longer cannot transmit the virus to others?” were categorised as “aware of U=U”.

### Statistical Analysis

Simple descriptive statistics (proportions, means, standard deviations and interquartile ranges) were used to describe the respondents' characteristics. The characteristics of those who experienced discrimination and those who did not were compared using Welch’s *t*-test and Pearson’s chi-squared test. Given the recognised threshold of 13 or higher on the K6 scale for identifying serious mental illness [23], the total score (ranging from 0 to 24) was dichotomised into two categories (0–12 and 13+) to represent the absence or presence of significant psychological distress in logistic regression analyses. This approach was chosen over treating each question on the K6 scale as a separate dependent variable. Simple and multiple logistic regression analyses were performed to investigate the association between HIV-related discrimination (independent variable) and the K6 scale (dependent variable, dichotomised based on a threshold score). After reviewing previous studies that suggested their association with mental health [24–26], the following variables were included as confounders: (i) age, (ii) regular psychiatric visits, and (iii) employment status. The interaction effect of HIV-related discrimination and HIV status disclosure on the K6 scale was first investigated using logistic regression analysis. This was done to assess how the interaction may influence the odds of experiencing serious mental illness. Additionally, linear regression analysis was employed to examine the more detailed impact of their interaction on the level of psychological distress. A log transformation of the K6 scale was not applied in the linear regression models, as residual distributions were relatively normal when observed.

## Results

### Overall Respondent Characteristics

In the sample set of 804 respondents, the median age was 46 years (interquartile range [IQR], 40–53) (see Table 1). Most respondents were male, and 70.0% (563/804) self-identified themselves as homosexual. A total of 11.9% (96/804) of respondents reported that they regularly visited a psychiatrist. While 16.8% (135/804) were currently unemployed, most respondents reported willingness to be in the workforce. As shown in Table 2, 49.8% (400/804) of respondents were diagnosed with HIV infection after 2011. A total of 78.0% (627/804) had undetectable viral loads, and 30.5% (245/804) reported that they had forgotten to take antiretroviral medicines according to their regimen at least once in the past month. The proportion of those who were aware of the U=U concept was 58.2% (468/804).

**Table 1:** Socio-demographics of respondents

**Characteristic**	**Total**		**Experience of HIV-related discrimination**				**Statistics**	***p*-value**^a^
			**Yes**		**No**			
	**(*n* = 804)**		**(*n* = 102)**		**(*n* = 702)**			
	***n***	**%**	***n***	**%**	***n***	**%**		
Age								
Mean	46.6	(SD = 10.1)	44.2	(SD = 9.9)	47.0	(SD = 10.1)	2.673	0.008
Median	46	(IQR, 40–53)	44	(IQR, 37–50)	47	(IQR, 41–53)		
20–29 years old	30	3.7%	3	2.9%	27	3.8%	13.854	0.007
30–39 years old	157	19.5%	33	32.4%	124	17.7%		
40–49 years old	325	40.4%	40	39.2%	285	40.6%		
50–59 years old	204	25.4%	18	17.6%	186	26.5%		
60 years old and above	88	10.9%	8	7.8%	80	11.4%		
Sex assigned at birth								
Male	771	95.9%	97	95.1%	674	96.0%	0.189	0.775
Female	33	4.1%	5	4.9%	28	4.0%		
Sexual orientation								
Homosexual	563	70.0%	70	68.6%	493	70.2%	1.091	0.777
Bisexual	124	15.4%	19	18.6%	105	15.0%		
Heterosexual	83	10.3%	9	8.8%	74	10.5%		
Others	34	4.2%	4	3.9%	30	4.3%		
Nationality								
Japanese	795	98.9%	99	97.1%	696	99.1%	3.503	0.094
Non-Japanese	9	1.1%	3	2.9%	6	0.9%		
University education								
Yes	361	44.9%	44	43.1%	317	45.2%	0.147	0.743
No	443	55.1%	58	56.9%	385	54.8%		
Visit psychiatrist regularly								
Yes	96	11.9%	25	24.5%	71	10.1%	17.553	< 0.001
No	708	88.1%	77	75.5%	631	89.9%		
Employment								
Employed	669	83.2%	76	74.5%	593	84.5%	6.327	0.018
Unemployed	135	16.8%	26	25.5%	109	15.5%		
Willingness to work								
Yes	751	93.4%	95	93.1%	656	93.4%	0.014	1.000
No	53	6.6%	7	6.9%	46	6.6%		

*SD* standard deviation, *IQR* interquartile range

^a^Monte Carlo simulation is applied with 2000 replicates. *p*-values smaller than 0.001 are all shown as < 0.001

**Table 2:** HIV-related characteristics of respondents

**Characteristic**	**Total**		**Experience of HIV-related discrimination**				**Statistics**	***p*-value**^a^
			**Yes**		**No**			
	**(*n* = 804)**		**(*n* = 102)**		**(*n* = 702)**			
	***n***	**%**	***n***	**%**	***n***	**%**		
Calendar year of HIV diagnosis								
2000 or before	82	10.2%	8	7.8%	74	10.5%	2.483	0.304
2001–2010	322	40.0%	36	35.3%	286	40.7%		
2011 onwards	400	49.8%	58	56.9%	342	48.7%		
CD4 count								
99 or less	37	4.6%	1	1.0%	36	5.1%	6.734	0.229
100–199	28	3.5%	4	3.9%	24	3.4%		
200–349	114	14.2%	10	9.8%	104	14.8%		
350–499	179	22.3%	28	27.5%	151	21.5%		
500 or more	417	51.9%	56	54.9%	361	51.4%		
Don’t know	29	3.6%	3	2.9%	26	3.7%		
Viral load								
Undetectable	627	78.0%	79	77.5%	548	78.1%	8.571	0.073
20–200	106	13.2%	20	19.6%	86	12.3%		
201–4999	14	1.7%	1	1.0%	13	1.9%		
5000 or more	13	1.6%	0	0.0%	13	1.9%		
Don’t know	44	5.5%	2	2.0%	42	6.0%		
Experience of developing AIDS								
Yes	225	28.0%	20	19.6%	205	29.2%	5.035	0.084
No	543	67.5%	75	73.5%	468	66.7%		
Don’t know	36	4.5%	7	6.9%	29	4.1%		
Forgotten use of antiretroviral medicines in the past month								
Yes	245	30.5%	40	39.2%	205	29.2%	4.215	0.045
No	559	69.5%	62	60.8%	497	70.8%		
Thought of excuses to hide HIV status								
Yes	547	68.0%	83	81.4%	464	66.1%	9.556	0.003
No	257	32.0%	19	18.6%	238	33.9%		
Pretended to be HIV-negative								
Yes	591	73.5%	88	86.3%	503	71.7%	9.778	0.003
No	213	26.5%	14	13.7%	199	28.3%		
Aware of U=U								
Yes	468	58.2%	64	62.7%	404	57.5%	0.988	0.347
No	336	41.8%	38	37.3%	298	42.5%		

*U*=*U* Undetectable=Untransmittable

^a^Monte Carlo simulation is applied with 2000 replicates. *p*-values smaller than 0.001 are all shown as < 0.001

When examining 252 respondents who were not included in the analysis due to their incomplete answers, it was observed that those who were excluded from the analysis were slightly older (median age, 49 years). Additionally, they were slightly less likely to be employed, express willingness to work, feign HIV status, and exhibit awareness of the U=U concept compared to the included group. No statistically significant differences were found between the two groups in any of the other characteristics.

### Characteristics of Those Who Experienced Discrimination

Of the 804 respondents, 12.7% (102/804) reported that they had recently experienced discrimination due to their HIV-positive status. The median age of those who experienced discrimination was 44 years (IQR, 37–50), whereas it was 47 years (IQR, 41–53) for those who did not (Table 1). While most socio-demographic characteristics were relatively similar between the two groups, those who experienced discrimination were more likely to visit psychiatrists regularly (*p* < 0.001) and currently unemployed (*p* < 0.05). As shown in Table 2, the percentage of those who forgotten to take antiretroviral medicines in the past month was significantly higher among those who experienced discrimination (39.2%, 40/102) than among those who did not (29.2%, 205/702). Those who experienced discrimination were also more likely to have thought of excuses to hide their HIV-positive status and pretended to be HIV-negative (*p* < 0.01).

### HIV Status Disclosure

Among the 804 respondents, 266 (33.1%) reported disclosing their HIV status to their parent(s) (Table 3). As the denominator includes those who do not have any parents, the figure can be interpreted as the proportion of respondents who have at least one parent aware of their HIV status. The proportions for sibling(s) and partner(s) were 26.4% (212/804) and 41.2% (331/804), respectively. 50.9% (409/804) of respondents reported not having any HIV-positive friends, while 35.1% (282/804) reported having HIV-positive friend(s) aware of their HIV status. A total of 29.6% (238/804) reported having other friend(s) aware of their HIV status. Figures for colleague(s) and boss(es) were much lower, at 4.6% (37/804) and 10.3% (83/804), respectively. 81.2% (653/804) reported having at least one person in the aforementioned relationship to whom they had disclosed their HIV status.

**Table 3:** HIV status disclosure (*n* = 804)

**Relationship**	**Disclosed HIV-positive status**					
	**Yes**		**No**		**No one of that relationship**	
	***n***	**%**	***n***	**%**	***n***	**%**
Parent(s)	266	33.1%	439	54.6%	99	12.3%
Sibling(s)	212	26.4%	507	63.1%	85	10.6%
Partner(s)	331	41.2%	122	15.2%	351	43.7%
HIV-positive friend(s)	282	35.1%	113	14.1%	409	50.9%
Other friend(s)	238	29.6%	460	57.2%	106	13.2%
Colleague(s)	37	4.6%	584	72.6%	183	22.8%
Boss(es)	83	10.3%	538	66.9%	183	22.8%

### Experience of Discrimination, HIV Status Disclosure, and Mental Health

As shown in Table 4, those who have experienced HIV-related discrimination had 2.61 higher odds (95% confidence interval [CI], 1.56–4.38) of having a K6 scale of 13 or higher, which is indicative of a serious mental illness. After adjusting for age, regular psychiatric visits, and employment status in the multiple logistic regression analysis, the experience of discrimination remained independently associated with a K6 scale of 13 or higher (adjusted odds ratio [aOR] 2.02; 95% CI, 1.15–3.57).

**Table 4:** Association between HIV-related discrimination experience and psychological distress (*n* = 804)

**Dependent variable**	**Experienced discrimination**		**Prevalence (%)**	**Simple**			**Multiple**		
				**OR**	**95% CI**	***p*-value**	**aOR**^**a**^	**95% CI**	***p*-value**
K6 scale	No	74/702	10.5%	Ref			Ref		
(0, < 13; 1, 13+)	Yes	24/102	23.5%	2.61	(1.56–4.38)	< 0.001	2.02	(1.15–3.57)	0.015

Model diagnosis for the multiple logistic regression model: the area under the curve (AUC) of the receiver operating characteristic (ROC) is 0.719. The Hosmer-Lemeshow test yields a chi-squared statistic of 0.448 with a corresponding *p*-value of 0.930 (number of bins = 5)

*CI* confidence interval, *aOR* adjusted odds ratio, *Ref* reference

^a^Adjusted for age, regular psychiatric visits, and employment status

The interaction effect (i.e. HIV-related discrimination × HIV status disclosure) was then added to the logistic regression model (Table 5). For all relationships, HIV status disclosure did not exert a significant interaction effect on the association between HIV-related discrimination and the K6 scale (binary variable). However, although not statistically significant, the aORs of less than 1 for the interaction effect indicate that disclosing one’s HIV status to partner(s), HIV-positive friend(s), and friend(s) partially moderated the odds of experiencing serious mental illness from HIV-related discrimination.

**Table 5:** Logistic regression analysis results (*n* = 804)

**Dependent variable = K6 scale (0, low; 1, high)**	**aOR**^a^	**95% CI**	***p*-value**^b^
Model 1 — HIV status disclosure to parent(s)/sibling(s)			
HIV-related discrimination (0, no; 1, yes)	1.46	(0.59–3.66)	0.415
HIV status disclosure to parent(s)/sibling(s) (0, no/no one of that relationship; 1, yes)	1.11	(0.66–1.87)	0.681
HIV-related discrimination × HIV status disclosure to parent(s)/sibling(s)	1.70	(0.53–5.46)	0.374
Model 2 — HIV status disclosure to partner(s)			
HIV-related discrimination (0, no; 1, yes)	2.50	(1.16–5.37)	0.019*
HIV status disclosure to partner(s) (0, no/no one of that relationship; 1, yes)	1.47	(0.89–2.43)	0.134
HIV-related discrimination × HIV status disclosure to partner(s)	0.62	(0.20–1.89)	0.400
Model 3 — HIV status disclosure to HIV-positive friend(s)			
HIV-related discrimination (0, no; 1, yes)	3.20	(1.60–6.41)	0.001**
HIV status disclosure to HIV-positive friend(s) (0, no/no one of that relationship; 1, yes)	0.90	(0.53–1.54)	0.710
HIV-related discrimination × HIV status disclosure to HIV-positive friend(s)	0.30	(0.09–1.00)	0.050
Model 4 — HIV status disclosure to friend(s)			
HIV-related discrimination (0, no; 1, yes)	2.61	(1.33–5.12)	0.005**
0.003 HIV status disclosure to friend(s) (0, no/no one of that relationship; 1, yes)	0.72	(0.41–1.29)	0.269
HIV-related discrimination × HIV status disclosure to friend(s)	0.48	(0.14–1.66)	0.244
Model 5 — HIV status disclosure to colleague(s)/boss(es)			
HIV-related discrimination (0, no; 1, yes)	1.98	(1.06–3.68)	0.032*
HIV status disclosure to colleague(s)/boss(es) (0, no/no one of that relationship; 1, yes)	0.98	(0.45–2.16)	0.965
HIV-related discrimination × HIV status disclosure to colleague(s)/boss(es)	1.15	(0.25–5.21)	0.856

*CI* confidence interval, *aOR*, adjusted odds ratio

^a^Adjusted for age, regular psychiatric visits, and employment status

^b^*p*-values smaller than 0.001 are all shown as < 0.001. *p < 0.05; **p < 0.01

The interaction effects of HIV status disclosure and HIV-related discrimination on psychological distress measured by the K6 scale (continuous variable) are shown in Table 6. As shown in model 3, disclosing one’s HIV status to HIV-positive friend(s) exerted a significant negative interaction on the association between HIV-related discrimination and the K6 scale (coefficient, −2.731; *p*-value = 0.010). A negative interaction effect of HIV-related discrimination and HIV status disclosure to other friend(s) on the K6 scale was also observed (coefficient, −3.115; *p*-value = 0.004). In other words, HIV status disclosure to HIV-positive friend(s) and other friend(s) both yielded positive effects of moderately buffering the association between HIV-related discrimination and psychological distress. The interaction effects were not significant for other relationships (i.e. parent(s)/sibling(s), partner(s), colleague(s)/boss(es)).

**Table 6:** Linear regression analysis results (*n* = 804)

**Dependent variable = K6 scale**	**Coefficient**^a^	**SE**	***t*** **value**	***p*-value**^b^
Model 1 — HIV status disclosure to parent(s)/sibling(s)				
HIV-related discrimination (0, no; 1, yes)	0.978	0.752	1.301	0.194
HIV status disclosure to parent(s)/sibling(s) (0, no/no one of that relationship; 1, yes)	−0.171	0.385	−0.445	0.657
HIV-related discrimination × HIV status disclosure to parent(s)/sibling(s)	1.051	1.046	1.005	0.315
Model 2 — HIV status disclosure to partner(s)				
HIV-related discrimination (0, no; 1, yes)	1.899	0.704	2.698	0.007**
HIV status disclosure to partner(s) (0, no/no one of that relationship; 1, yes)	0.255	0.378	0.675	0.500
HIV-related discrimination × HIV status disclosure to partner(s)	−0.894	1.047	−0.854	0.394
Model 3 — HIV status disclosure to HIV-positive friend(s)				
HIV-related discrimination (0, no; 1, yes)	2.635	0.683	3.858	< 0.001***
HIV status disclosure to HIV-positive friend(s) (0, no/no one of that relationship; 1, yes)	0.226	0.393	0.575	0.566
HIV-related discrimination × HIV status disclosure to HIV-positive friend(s)	−2.731	1.056	−2.587	0.010**
Model 4 — HIV status disclosure to friend(s)				
HIV-related discrimination (0, no; 1, yes)	2.665	0.664	4.011	< 0.001***
HIV status disclosure to friend(s) (0, no/no one of that relationship; 1, yes)	0.634	0.416	1.525	0.128
HIV-related discrimination × HIV status disclosure to friend(s)	−3.115	1.073	−2.901	0.004**
Model 5 — HIV status disclosure to colleague(s)/boss(es)				
HIV-related discrimination (0, no; 1, yes)	1.545	0.583	2.648	0.008**
HIV status disclosure to colleague(s)/boss(es) (0, no/no one of that relationship; 1, yes)	0.128	0.600	0.214	0.831
HIV-related discrimination × HIV status disclosure to colleague(s)/boss(es)	−0.231	1.386	−0.167	0.868

*SE* standard error

^a^Adjusted for age, regular psychiatric visits, and employment status

^b^*p*-values smaller than 0.001 are all shown as < 0.001. **p < 0.01; ***p < 0.001

## Discussion

In this study, more than one-tenth of 804 PLWH in Japan infected through sexual contact experienced HIV-related discrimination. In addition, the findings of this study corroborate the results of a previous study in which HIV-related discrimination was associated with poor mental health among PLWH [27]. The most noteworthy finding of this study is the detection of a significant negative interaction between disclosing one’s HIV status to friends and HIV-related discrimination on psychological distress, as demonstrated by the linear regression analysis. This finding suggests that having friends who are aware of one’s HIV status could weaken the impact of HIV-related discrimination on psychological distress among PLWA in Japan. This underscores the potential benefits of interventions designed to support PLWA in making informed decisions about disclosing their HIV status, with the goal of safeguarding their mental health from the adverse effects of discrimination.

One possible hypothesis for this finding is social support theory, in which HIV status disclosure facilitates social support that protects PLWH from the negative effects of stressful events [28]. Substantial evidence suggests that loneliness and poor social support are associated with poorer depression outcomes [29]. Although the level of perceived social support was not quantitatively measured, the findings of this study suggest that PLWH who disclose their HIV status to friends are likely to receive increased social support and security, which in turn leads to improved self-esteem and greater ability to cope with the negative consequences of HIV-related discrimination. Less than 30% of respondents in this study had friends who were aware of their HIV status, suggesting an opportunity to enhance social support for PLWH by encouraging HIV status disclosure to trusted friends. However, as disclosure of HIV status also entails potential risks, such as heightened vulnerability to internalised stigma [30] and the possibility of facing rejection and losing friends and family [17], it is important that PLWH are provided with a conducive environment that supports when, how, and to whom they disclose their HIV-positive status, in order to optimise the benefits of HIV status disclosure.

On the other hand, this study did not observe a significant protective influence of disclosing one’s HIV status to family members on the association between discrimination experience and psychological distress. This implies that HIV status disclosure to family members, unlike friends, does not necessarily facilitate social support and better mental health among PLWH. A previous study conducted in the United States also reported that HIV-positive youths who disclosed their HIV-positive status to at least one family member did not differ in their levels of perceived social support from those who chose not to disclose their serostatus to their families [28]. The results of this study offer insights into the significance and implications of disclosing HIV status to family members among PLWH in Japan. First, in Asian Confucian and collectivistic-oriented cultures in healthcare settings, where family members are often involved in HIV diagnosis and treatment processes [31], disclosing one’s HIV status to family members may be seen as an obligation rather than a sign of help-seeking. Hence, it is plausible that PLWH in Japan may not necessarily seek emotional support from family members upon disclosing their HIV status, leading to a lack of improvement in social support and mental health.

Moreover, it is important to note that the disclosure of HIV-positive status to family members often coincides with the disclosure of their sexual orientation, as gay, bisexual, and other men who have sex with men (gbMSM) contribute to a significant proportion of new HIV infections in Japan [19]. In contemporary Japanese society, the family unit is frequently perceived as the source of homophobia, where negative sentiments and attitudes towards homosexuality are expressed [32]. This stems in part from a societal pressure for conformity to traditional norms, leading parents to harbour fears of public scrutiny and potentially react unfavourably to children deviating from heteronormative expectations [33]. Therefore, even if an individual expects assistance from family after disclosing their HIV-positive status, it is likely that they may not receive adequate emotional support from parents, who are feeling uncomfortable upon discovering that their child is homosexual. In contrast, friends, who exist outside the confines of familial expectations, may offer a more accepting environment and different perspective, thereby enhancing self-esteem in PLWH and protecting them from psychological distress.

Our findings should be considered within the context of several limitations. First, the respondents were recruited from eight leading HIV/AIDS care hospitals in Japan; thus, the findings may not necessarily be generalisable to all PLWH in Japan. In addition, participation bias may have impacted the external validity of the findings. The recruitment process was structured in accordance with the order of outpatient visits, potentially leading to a higher likelihood of participation among individuals with shorter intervals between visits, typically those recently diagnosed. It is also noteworthy that slightly older patients were more likely to have incomplete responses, implying that non-responders (i.e. patients who did not return the questionnaire despite consenting) may skew towards an older age group. This indicates a potential bias towards younger, healthier patients in the analytical samples. This is further corroborated by the exclusion of patients with poor physical health from the study. Moreover, self-reported data may have caused several reporting biases, although the complete anonymity of the questionnaire may have minimised socially desirable responses. Lastly, the study could not infer causation from the associations observed owing to its cross-sectional design. Therefore, it is also possible to interpret that better mental health may have facilitated some PLWH to disclose their HIV-positive status, and not necessarily vice versa.

## Conclusion

In conclusion, this study, which derived data from a nationwide health and lifestyle survey of PLWH in Japan, found that more than one-tenth of respondents reported experiencing HIV-related discrimination. Notably, those who faced HIV-related discrimination exhibited higher levels of psychological distress, with the disclosure of their HIV status to friends partially mitigating this association. Discrimination should not be tolerated and continuous efforts must be made to ensure that PLWH can live without fear of stigma and discrimination. At the same time, assessing individual needs of PLWH for social support and increasing their opportunities to communicate with trusted friends whom they feel comfortable disclosing their HIV status may be equally important to facilitate social support and protect their mental health from the detrimental effects of discrimination.

## Data Availability

The data that support this study cannot be publicly shared due to privacy reasons, but may be shared upon reasonable request to the corresponding author, if appropriate.

## Declarations

### Research Involving Animals

This article does not contain any studies with animals performed by any of the authors.

## References

[1] Marcus JL, Leyden WA, Alexeeff SE, et al. Comparison of overall and comorbidity-free life expectancy between insured adults with and without HIV infection, 2000–2016. JAMA Netw Open. 2020;3(6):e207954. 32539152 10.1001/jamanetworkopen.2020.7954 PMC7296391

[2] Remien RH, Stirratt MJ, Nguyen N, Robbins RN, Pala AN, Mellins CA. Mental health and HIV/AIDS: the need for an integrated response. AIDS. 2019;33(9):1411–20. 30950883 10.1097/QAD.0000000000002227 PMC6635049

[3] Niu L, Luo D, Liu Y, Silenzio VM, Xiao S. The mental health of people living with HIV in China, 1998–2014: a systematic review. PLoS ONE. 2016;11(4):e0153489. 27082749 10.1371/journal.pone.0153489 PMC4833336

[4] Hikasa S, Shimabukuro S, Hideta K, et al. Quality of life of people living with HIV compared with that of the general population in Japan. J Infect Chemother. 2017;23(10):698–702. 28811073 10.1016/j.jiac.2017.07.012

[5] Uthman OA, Magidson JF, Safren SA, Nachega JB. Depression and adherence to antiretroviral therapy in low-, middle- and high-income countries: a systematic review and meta-analysis. Curr HIV/AIDS Rep. 2014;11(3):291–307. 25038748 10.1007/s11904-014-0220-1 PMC4359613

[6] Shrestha R, Copenhaver M, Bazazi AR, Huedo-Medina TB, Krishnan A, Altice FL. A moderated mediation model of HIV-related stigma, depression, and social support on health-related quality of life among incarcerated Malaysian men with HIV and opioid dependence. AIDS Behav. 2017;21(4):1059–69. 28108877 10.1007/s10461-017-1693-x PMC5344717

[7] Omiya T, Yamazaki Y, Shimada M, et al. Mental health of patients with human immunodeficiency virus in Japan: a comparative analysis of employed and unemployed patients. AIDS Care. 2014;26(11):1370–8. 24839867 10.1080/09540121.2014.913765

[8] Bavinton BR, Pinto AN, Phanuphak N, et al. Viral suppression and HIV transmission in serodiscordant male couples: an international, prospective, observational, cohort study. Lancet HIV. 2018;5(8):e438–47. 30025681 10.1016/S2352-3018(18)30132-2

[9] Togari T, Abe S, Inoue Y. HIV-related public stigma and knowledge regarding the campaign slogan “undetectable=untransmittable” among Japanese people. Nihon Koshu Eisei Zasshi. 2022;69(2):146–57. 34924493 10.11236/jph.21-005

[10] Yu F, Hsiao YH, Park S, et al. The influence of anticipated HIV stigma on health-related behaviors, self-rated health, and treatment preferences among people living with HIV in East Asia. AIDS Behav. 2023;27(4):1287–303. 36348191 10.1007/s10461-022-03865-5 PMC10036452

[11] Komatsu K, Kimura S, Kiryu Y, Oka S, Takahashi H, Matsushima E, et al. Detailed analysis of social support and proactive coping with depressive symptoms in Japanese HIV-infected individuals. AIDS Care. 2022;34(8):1022–30. 34082633 10.1080/09540121.2021.1934382

[12] Zhang Y, Chai C, Xiong J, et al. The impact of anxiety, depression, and social support on the relationship between HIV-related stigma and mental health-related quality of life among Chinese patients: a cross-sectional, moderate-mediation study. BMC Psychiatry. 2023;23(1):818. 37940853 10.1186/s12888-023-05103-1 PMC10634100

[13] Casale M, Boyes M, Pantelic M, Toska E, Cluver L. Suicidal thoughts and behaviour among South African adolescents living with HIV: can social support buffer the impact of stigma? J Affect Disord. 2019;245:82–90. 30368074 10.1016/j.jad.2018.10.102

[14] Armoon B, Fleury MJ, Bayat AH, et al. HIV related stigma associated with social support, alcohol use disorders, depression, anxiety, and suicidal ideation among people living with HIV: a systematic review and meta-analysis. Int J Ment Health Syst. 2022;16(1):17. 35246211 10.1186/s13033-022-00527-w PMC8896327

[15] Yao TY, Liou BH, Chien WC, Wu FL. Disclosure concerns and the correlation among gay, bisexual, and other men who have sex with men living with HIV receiving antiretroviral therapy in Taiwan. Health Serv Insights. 2024;17:11786329231224620. 38264172 10.1177/11786329231224620 PMC10804901

[16] Gabbidon K, Chenneville T, Peless T, Sheared-Evans S. Self-disclosure of HIV status among youth living with HIV: a global systematic review. AIDS Behav. 2020;24(1):114–41. 30924065 10.1007/s10461-019-02478-9

[17] Lin X, Chi P, Zhang L, Zhang Y, Fang X, Qiao S, et al. Disclosure of HIV serostatus and sexual orientation among HIV-positive men who have sex with men in China. Community Ment Health J. 2016;52(4):457–65. 26002087 10.1007/s10597-015-9879-z PMC6234002

[18] Serovich JM, Laschober TC, Brown MJ, Kimberly JA, Lescano CM. Effects of a decision-making intervention to help decide whether to disclose HIV-positive status to family members on well-being and sexual behavior. Arch Sex Behav. 2020;49(6):2091–101. 32328912 10.1007/s10508-020-01703-0 PMC7321873

[19] Ichikawa S. The current situation of HIV/AIDS among MSM (men who have sex with men) in Japan—from the viewpoint of socio-epidemiology. AIDS Res. 2017;19(2):71–80.

[20] Swinbanks D. Documents confirm delays in Japanese HIV blood scandal. Nature. 1996;379(6568):760. 8587591 10.1038/379760a0

[21] Oka S, Ikeda K, Takano M, et al. Pathogenesis, clinical course, and recent issues in HIV-1-infected Japanese hemophiliacs: a three-decade follow-up. Glob Health Med. 2020;2(1):9–17. 33330768 10.35772/ghm.2019.01030 PMC7731362

[22] Furukawa TA, Kawakami N, Saitoh M, et al. The performance of the Japanese version of the K6 and K10 in the World Mental Health Survey Japan. Int J Methods Psychiatr Res. 2008;17(3):152–8. 18763695 10.1002/mpr.257 PMC6878390

[23] Kessler RC, Andrews G, Colpe LJ, et al. Short screening scales to monitor population prevalences and trends in non-specific psychological distress. Psychol Med. 2002;32(6):959–76. 12214795 10.1017/s0033291702006074

[24] Paul KI, Moser K. Unemployment impairs mental health: meta-analyses. J Vocat Behav. 2009;74(3):264–82.

[25] Pieh C, Budimir S, Probst T. The effect of age, gender, income, work, and physical activity on mental health during coronavirus disease (COVID-19) lockdown in Austria. J Psychosom Res. 2020;136:110186. 32682159 10.1016/j.jpsychores.2020.110186 PMC7832650

[26] Hossain MM, Tasnim S, Sultana A, et al. Epidemiology of mental health problems in COVID-19: a review. F1000Res. 2020;9:636. 33093946 10.12688/f1000research.24457.1 PMC7549174

[27] Earnshaw VA, Eaton LA, Collier ZK, et al. HIV stigma, depressive symptoms, and substance use. AIDS Patient Care STDS. 2020;34(6):275–80. 32484742 10.1089/apc.2020.0021 PMC7262648

[28] Lee S, Yamazaki M, Harris DR, Harper GW, Ellen J. Social support and human immunodeficiency virus-status disclosure to friends and family: implications for human immunodeficiency virus-positive youth. J Adolesc Health. 2015;57(1):73–80. 25940217 10.1016/j.jadohealth.2015.03.002 PMC4478132

[29] Wang J, Mann F, Lloyd-Evans B, Ma R, Johnson S. Associations between loneliness and perceived social support and outcomes of mental health problems: a systematic review. BMC Psychiatry. 2018;18(1):156. 29843662 10.1186/s12888-018-1736-5 PMC5975705

[30] Mugo C, Seeh D, Guthrie B, et al. Association of experienced and internalized stigma with self-disclosure of HIV status by youth living with HIV. AIDS Behav. 2021;25(7):2084–93. 33389374 10.1007/s10461-020-03137-0 PMC8768004

[31] Chen WT, Starks H, Shiu CS, et al. Chinese HIV-positive patients and their healthcare providers: contrasting Confucian versus Western notions of secrecy and support. ANS Adv Nurs Sci. 2007;30(4):329–42. 18025868 10.1097/01.ANS.0000300182.48854.65 PMC3583193

[32] Tamagawa M. Same-sex marriage in Japan. J GLBT Fam Stud. 2016;12(2):160–87.

[33] Tamagawa M. Coming out of the closet in Japan: an exploratory sociological study. J GLBT Fam Stud. 2018;14(5):488–518.

